# Diagnostic accuracy of code-free deep learning for detection and evaluation of posterior capsule opacification

**DOI:** 10.1136/bmjophth-2022-000992

**Published:** 2022-05-23

**Authors:** Josef Huemer, Martin Kronschläger, Manuel Ruiss, Dawn Sim, Pearse A Keane, Oliver Findl, Siegfried K Wagner

**Affiliations:** 1 Department of Medical Retina, Moorfields Eye Hospital NHS Foundation Trust, London, UK; 2 NIHR Biomedical Research Centre, Moorfields Eye Hospital NHS Foundation Trust and UCL Institute of Ophthalmology, London, UK; 3 VIROS-Vienna Institute for Research in Ocular Surgery, a Karl Landsteiner Institute, Hanusch Hospital, Vienna, Austria; 4 Institute of Ophthalmology, UCL, London, UK

**Keywords:** diagnostic tests/investigation, imaging, lens and zonules

## Abstract

**Objective:**

To train and validate a code-free deep learning system (CFDLS) on classifying high-resolution digital retroillumination images of posterior capsule opacification (PCO) and to discriminate between clinically significant and non-significant PCOs.

**Methods and analysis:**

For this retrospective registry study, three expert observers graded two independent datasets of 279 images three separate times with no PCO to severe PCO, providing binary labels for clinical significance. The CFDLS was trained and internally validated using 179 images of a training dataset and externally validated with 100 images. Model development was through Google Cloud AutoML Vision. Intraobserver and interobserver variabilities were assessed using Fleiss kappa (κ) coefficients and model performance through sensitivity, specificity and area under the curve (AUC).

**Results:**

Intraobserver variability κ values for observers 1, 2 and 3 were 0.90 (95% CI 0.86 to 0.95), 0.94 (95% CI 0.90 to 0.97) and 0.88 (95% CI 0.82 to 0.93). Interobserver agreement was high, ranging from 0.85 (95% CI 0.79 to 0.90) between observers 1 and 2 to 0.90 (95% CI 0.85 to 0.94) for observers 1 and 3. On internal validation, the AUC of the CFDLS was 0.99 (95% CI 0.92 to 1.0); sensitivity was 0.89 at a specificity of 1. On external validation, the AUC was 0.97 (95% CI 0.93 to 0.99); sensitivity was 0.84 and specificity was 0.92.

**Conclusion:**

This CFDLS provides highly accurate discrimination between clinically significant and non-significant PCO equivalent to human expert graders. The clinical value as a potential decision support tool in different models of care warrants further research.

**What is already known on this topic?**

- Deep learning (DL) has been proven to be a powerful tool for image analysis and has been applied to cataract-related image classification. Posterior capsule opacification (PCO) can be detected by retroillumination images and is the most common complication of cataract surgery.

**What this study adds?**

- Code-free DL can be used to train DL systems to detect clinically significant PCO. Clinicians can use code-free DL with little coding experience to develop clinically relevant artificial intelligence applications.

**How this study might affect research, practice or policy?**

- This novel use case of code-free DL explores new areas of clinical relevance outside of the classic domains of DL in ophthalmology and serves as a proof of concept to help bridge the gap between research and potential clinical applications.

## Introduction

The recent progress in artificial intelligence (AI) is mainly attributed to the development of deep learning (DL), a subdivision of machine learning, with major improvements in the diagnostic performance of image recognition, speech recognition and natural language processing.^1^ Its use in medicine in particular has been shown to perform on par with humans in imaging-based specialities like radiology, dermatology and ophthalmology.^2^ Whereas traditional DL relies heavily on vast computing power and coding skills, recent developments of automated, code-free neural networks using transfer learning or neural architecture search have allowed clinicians to investigate datasets independently and to reproduce previously achieved results like predicting sex from colour fundus photographs.^3 4^

Formation of cataract is the leading cause of treatable blindness, with surgical lens removal as the only option of treatment.^5^ Multiple aspects of cataracts and the respective surgery have been analysed with AI, including screening and grading of colour slit lamp photographs, optimisation of preoperative intraocular lens (IOL) calculations and posterior capsule opacification (PCO) prediction.^6–8^ The most common complication after cataract surgery with IOL implantation is the development of PCO.^9 10^ Incidence of PCO ranges from <5% to 50%^11^ and recently was reported for monofocal single-piece IOLs to range between 7.1% and 22.6% at 5 years.^12^ The most common effective treatment of PCO is neodymium-doped yttrium aluminium garnet laser capsulotomy, which occasionally involves the following complications: elevated intraocular pressure, retinal detachment and endophthalmitis.^13^ Therefore, evaluation of clinically significant versus non-significant PCO is of clinical relevance.

The aim of this study was to investigate a code-free deep learning system (CFDLS) trained to detect clinically significant PCO on retroillumination photography and to compare its outcome to human expert graders.

## Materials and methods

This study was in compliance with the Declaration of Helsinki andreporting guidelines for diagnostic accuracy, the Standards for Reporting of Diagnostic Accuracy (STARD).^14^

### Study design

This was a retrospective study using previously acquired data as part of a prospective observation cohort.^15^ The optical system at the time of recording consisted of a Zeiss 30-slit lamp for observation and imaging, a Zeiss retrolux illumination module with illumination provided by a Zeiss anterior segment flash pack through a fibre-optic cable and beam splitters. A Kodak NC2000 digital camera with high light sensitivity resulting from a 16.0 mm×21.0 mm charge-coupled device (CCD) chip was used, resulting in a high signal-to-noise ratio in the acquired images. The CCD had a geometric resolution of 1268 pixels×1012 pixels and a radiometric resolution of 36 bits (red, green and blue). The images were directly imported into Adobe Photoshop V.5.5 and saved to a hard disk in tagged image file format (TIFF, 3.85 megabytes per image).^15^

**Figure 1:**
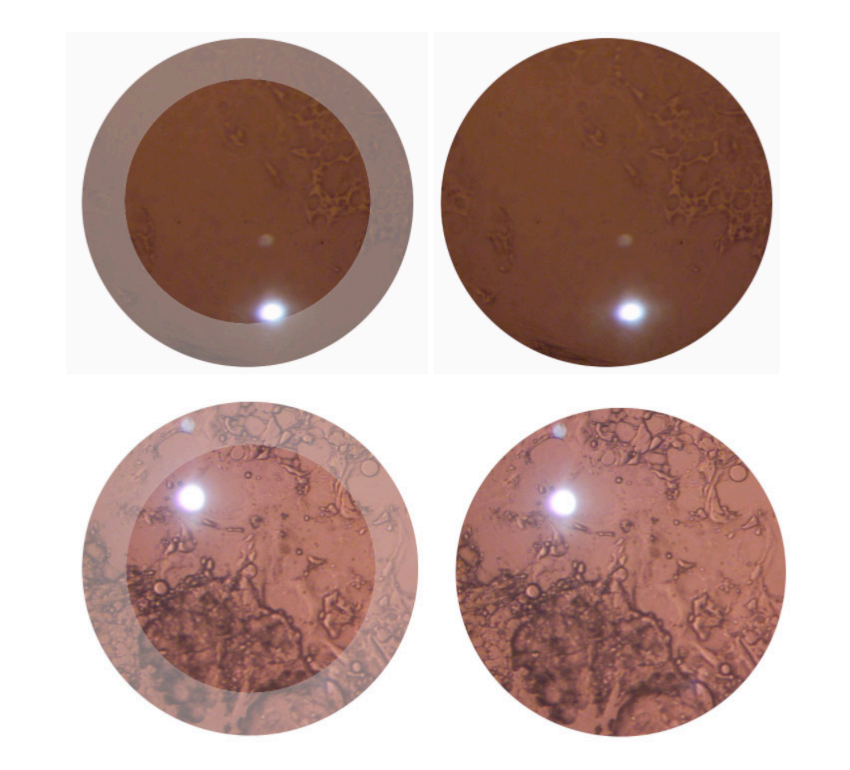
Examples of non-significant (above) and significant (below) posterior capsule opacification with central 3 mm region of interest highlighted on the left side only available for the human expert graders.

The region of interest (ROI) was defined as the central 4 mm of the IOL not containing any structures of the anterior capsule. This was accomplished by importing the images into Gimp V.2.10.14, an open-source cross-platform imaging editor, and manually cropping the images (figure 1). Patient-identifying information was not accessible.

### Datasets

The training dataset consists of 179 images with various grades of PCO, containing at least 12 images per grade and is described in detail in table 1. Random partition of the dataset into training, tuning and test (internal validation) was automatically implemented by the Google Cloud AutoML Vision application programming interface (API) in an 80–10–10 distribution.

**Table 1:** Distribution of classes in the development and external validation datasets

	Development			External
	Train	Validation	Test	Test
Non-significant	67	9	8	37
Significant	76	10	9	63
Total	143	19	17	100

To perform an external validation,^16^ a set of 100 digital images of eyes of 100 patients with an even distribution of mild to severe PCO manually selected by an experienced examiner for a previous study^15^ was used; patient-identifying information was not accessible (table 1). All images had been imported to Adobe Photoshop V.5.5 and processed as TIFF files in 2002 in a similar fashion as the training set. The external validation dataset was created to assess quality assurance measures in 2002; the training dataset consisted of images taken at the same institution between 2005 and 2008.

### Grading

Labels were defined in a binary fashion as clinically non-significant or significant. An opacification in the central 3 mm, previously defined as the most significant area,^17^ was determined to be significant; examples of clinical grades can be seen in figure 1.

For both training dataset and validation dataset, three sets of data in a random sequence were generated, respectively, using an online randomisation system (https://www.randomizer.org). All three sets of each training dataset and validation dataset were presented to three board-certified ophthalmologists and experienced cataract surgeons. The investigators graded the images completely independent from each other and were masked to the results of each other. The final grading of each grader was determined by the majority vote of the three votes from the same grader for each image.

### Development of the DL algorithm

Whereas DL usually requires advanced coding knowledge and intensive computing power using multiple graphical processing units, recent developments of automated neural networks allow clinicians with little coding skills to investigate datasets with AI.^3 4^ These techniques depend on transfer learning (using previously trained algorithms for different purposes to retrain for a new task) and neural architecture search (a technique of automatic neural network architecture selection). APIs are available by multiple providers. In this study, we leveraged the Google Cloud AutoML Vision API (Google). Anonymised datasets are uploaded through graphical user interfaces (GUIs) in the API onto a cloud bucket for the training and validation process.^18^ Repeated images were removed and the datasets were handled separately to avoid overfitting. Due to the architecture of the APIs, different experiments were performed for comparability.

### Statistical analysis

Intraobserver and interobserver variabilities of categorical variables (significant vs non-significant PCO) were assessed using the Fleiss kappa (κ) statistic for categorical results by multiple graders as described by Landis and Koch with 95% bootstrap CIs estimated through Monte Carlo simulations using 1000 iterations.^19^

Model performance was through sensitivity, specificity and area under the curve (AUC) with 95% CIs estimated using 2000 stratified bootstrap replicates. Where appropriate, fourfold confusion matrices for internal and external validations and receiver operating characteristic (ROC) curves are shown. All analyses were conducted in R V.4.1.0 (R Core Team, R Foundation for Statistical Computing, Vienna, Austria) with the caret, pROC and raters package for analysis and ggplot for visualisations.

## Results

The development dataset consisted of 179 images, 67 of which were without or with non-significant PCO and 76 significant PCOs (table 1). Intraobserver variability κ (95% CI) for the three gradings for observers 1, 2 and 3 were 0.90 (95% CI 0.86 to 0.95), 0.94 (95% CI 0.90 to 0.97) and 0.88 (95% CI 0.82 to 0.93), respectively. Interobserver κ for the final grading for all three observers was 0.84 (95% CI 0.78 to 0.89) and that for all nine gradings was 0.82 (95% CI 0.76 to 0.86). Pairwise comparisons between each observer as well as the majority vote are shown in table 2. Interobserver agreement was generally high, ranging from 0.85 (95% CI 0.79 to 0.90) between observers 1 and 2 to 0.90 (95% CI 0.85 to 0.94) for observers 1 and 3.

**Table 2:** Fleiss κ between observers and majority vote

	Observer 1	Observer 2	Observer 3	Majority vote
Observer 1	X			
Observer 2	0.85 (0.79 to 0.90)	X		
Observer 3	0.90 (0.85 to 0.94)	0.88 (0.82 to 0.93)	X	
Majority vote	0.93 (0.87 to 0.97)	0.93 (0.88 to 0.96)	0.96 (0.92 to 0.99)	X

**Figure 2:**
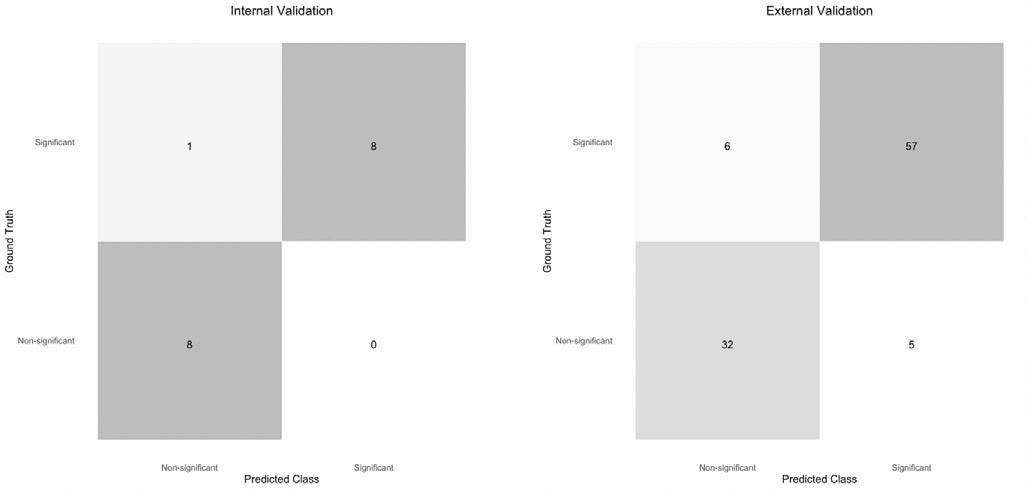
Fourfold confusion matrices for the internal validation and external validation sets.

**Figure 3:**
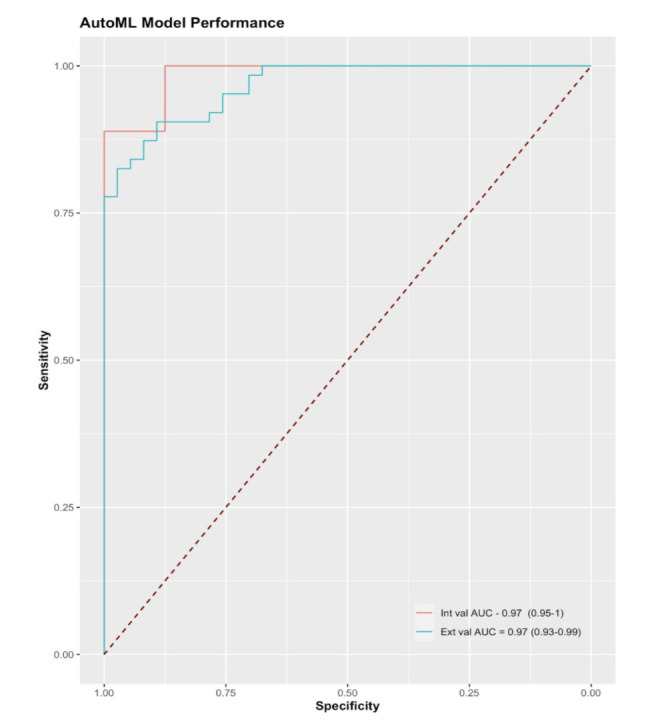
Receiver operating characteristic curve showing model performance on the internal and external validation test sets across different thresholds. The boundary of no discrimination is shown in a dotted red line. AUC, area under the curve.

Fourfold confusion matrices for the internal and external validation sets are shown in figure 2. On the internal validation dataset, sensitivity was 0.89 at a specificity of 1 and the AUC was 0.9861 (95% CI 0.92 to 1.0). The external validation dataset consisted of 100 images, of which 63 were visually significant PCOs. On external validation, sensitivity was 0.84 and specificity was 0.92. The AUC was 0.9661 (95% CI 0.93 to 0.99). ROC curves for the internal and external validation datasets are shown in figure 3.

### Error auditing

Qualitative review of algorithmic misclassifications were carried out by the three observers. In the cases where the CFDLS predicted the PCO to be false positively significant, peripheral PCO outside the 3 mm ROI could be observed. When analysing the cases, the DLS wrongly predicted as non-significant, two things were striking: first, none of the cases presented with pearls, and in the majority of cases, posterior capsule folds could be observed (online supplemental figure 1).

10.1136/bmjophth-2022-000992.supp1 Supplementary data

## Discussion

In this study, we developed and validated a CFDLS classifying between clinically significant and non-significant PCO in retroillumination images. The CFDLS showed a robust performance in detecting clinically significant PCO with a sensitivity of 0.84, a specificity of 0.92 and an AUC of 0.9661 (95% CI 0.93 to 0.99) on external validation. This proof of concept shows that CFDLS can be used to develop potential decision support tools and enables clinicians to expand into the clinical research of AI and explore novel use cases of AI applications.

The validation dataset was created in 2002 and consists of images with different degrees of PCO. Findl *et al* already used the dataset for comparison of four methods (subjective analysis, Evaluation of Posterior Capsule Opacification [EPCO], Posterior Capsule Opacity [POCO] and Automated Quantification of After-Cataract [AQUA] I studies) of PCO quantification.^15^ Moreover, Kronschläger *et al* applied the same dataset in creating an automated qualitative and quantitative assessment tool of PCO, that is, AQUA II.^20^ AQUA II already showed excellent validity and repeatability. Projecting light into the eye causes Purkinje spots on each tissue interface. Additionally, internal reflections of the optics of the system used may appear. Those light spots and reflections (figure 1) on the image cover pathological changes of the posterior capsule and therefore are missing in the grading process. Because the Purkinje spots change their position in slightly different directions of gaze, merging of images of different gaze positions enables the removal of light-reflection artefacts.^21^ However, at the time of data collection, this method was yet not published.

A rigorous and sound grading process to establish a ground truth is especially important when labels are provided to develop DL classifiers.^22^ Krause *et al* demonstrated the importance of arbitration to improve the algorithmic performance for diabetic retinopathy grades.^23^ Whereas previous grading approaches for PCO focused on quantitative human grading,^15^ for this study, we decided to proceed with a binary grading that was aiming to label clinical significance. The rationale behind this is based on the visual significance of the inner area of 3 mm; binary labels were used to reduce the risk of PCO underestimation.^24^ Good intraobserver and interobserver variabilities were achieved by the three expert graders.

Applications using AI are heading towards all fields of medicine. A recent survey from the American College of Radiology showed that 30% of radiologists were using AI in some form in their clinical practice.^25^ Teleophthalmology may serve as a solution to increasing demands and stretched services in the field of cataract surgery.^8^ Wu *et al* have presented results of a universal AI model for a collaborative management of cataract, with referral decisions for preoperative and postoperative grading, requiring a large dataset for training and bespoke modular architectures.^8^ The model performance in our study in the external validation was respectable, with a sensitivity of 0.84 and a specificity of 0.92, with an AUC of 0.9661, warranting further research using larger datasets.

With CFDLS, clinicians now have the opportunity to explore clinical datasets using cloud-based APIs and GUIs. The ability to understand the complexity of clinical data in combination with code-free platforms will allow clinicians to further explore clinical use cases. Although little coding skills are required to train such bespoken models, the process of data preparation remains to be a major part of such studies. Furthermore, clinicians need to have a good understanding of the importance of labelling, grading, training, well-balanced distributions and potential hidden confounders when developing CFDLS.^26^ Automated CFDLSs have been shown to perform comparably with bespoke classifiers in ophthalmology and other fields of medicine. On the other hand, lack of adjustable model architectures during training as well as the ‘black box’ phenomena may serve as limitations.^27^ As explainability methods still remain to be challenging, Ghassemi *et al* have argued that rigorous internal and external validations serve as a more achievable goal to evaluate the performance of DL systems.^28^ Classifiers as developed in this study could help to exclude PCO in triage settings and could be externalised into smartphone-based home screening applications. Once revalidated, it may serve as a decision support tool in a referral refinement process. This proof of concept shows that clinicians can use AI to explore novel applications in ophthalmology outside the classic domains of retinal imaging and glaucoma.

Error auditing showed interestingly that peripheral PCO was noted in the cases to be predicted as false positive. This could be refined by first incorporating a preprocessing step of peripheral cropping. Second, formal occlusion testing of the periphery would bolster this justification but was outside the remit of this project. The importance of error auditing in AI cannot be underestimated to identify and prevent algorithmic bias both inside and outside of healthcare.^29 30^

The limitations of our study include the size of the datasets and the setting of a single centre with a mainly Caucasian population. No multifocal lenses were included in the dataset as the curation predated multifocal IOLs. The model design of the CFDLS in terms of model architecture and hyperparameters is not transparent; it has the potential to diminish machine learning explainability even further due to a lack of understanding of the model architectures and parameters used. Preprocessing of the images limits the scalability but could be incorporated in a more user-friendly application prior to incorporation. To explore generalisation, further evaluation using a larger dataset representing a multiethical population therefore is warranted.

In conclusion, we trained a CFDLS to classify between significant and non-significant PCO on retroillumination images with high sensitivity and specificity. Moreover, the CFDLS equaled human expert graders in reliability. This CFDLS for PCO serves as proof of concept to support the decision whether PCO needs to be addressed by yttrium aluminium garnet capsulotomy, possibly even in a teleophthalmological or triage setting.

## Data Availability

Data are available upon request. Interested parties should contact JH.
