# Supplementary figures and images for: Diagnostic accuracy of code-free deep learning for detection and evaluation of posterior capsule opacification
Source: BMJ Open Ophthalmol. 2022 May 23;7(1):e000992. doi: 10.1136/bmjophth-2022-000992 (PMC9174773; doi:10.1136/bmjophth-2022-000992)

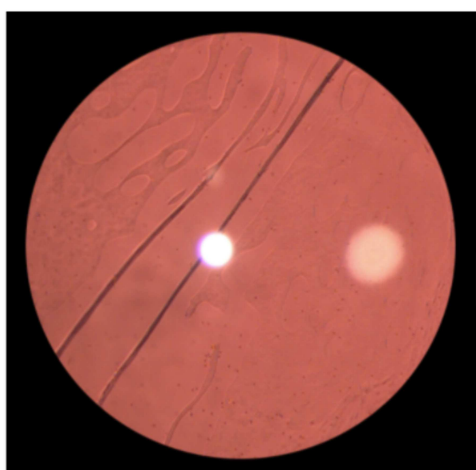

GT Significant - majority vote  
Prediction: Non-significant  
7.316.023

Supplement: Supplementary data [file bmjophth-2022-000992supp001.pdf]
